# FESEM evaluation of smear layer removal from conservatively shaped canals: laser activated irrigation (PIPS and SWEEPS) compared to sonic and passive ultrasonic activation—an ex vivo study

**DOI:** 10.1186/s12903-021-01427-0

**Published:** 2021-02-22

**Authors:** Manuele Mancini, Loredana Cerroni, Pietro Palopoli, Giovanni Olivi, Matteo Olivi, Cristiano Buoni, Luigi Cianconi

**Affiliations:** 1 Department of Translational Medicine and Clinical Science, University of Rome “Tor Vergata”, Via Montpellier 1, 00133 Rome, Italy; 2 Catholic University of the Sacred Hearth of Rome, Rome, Italy; 3 Private Practice, Rome, Italy; 4 Department of Surgical Sciences, Dental School, Endodontics, University of Turin, 10126 Turin, Italy

**Keywords:** Smear layer removal, Conservative canal shaping, Sonic activation, PUI, PIPS, SWEEPS

## Abstract

**Background:**

Irrigation of the pulp space is a mandatory step to get rid of all its organic and inorganic content. Activation of the irrigants play a key role in the era of minimally invasive endodontics. The aim of this study was to assess the effectiveness of different irrigants activation methods in removing the smear layer at 1, 3, 5 and 8 mm from the apex from conservatively shaped canals.

**Methods:**

Eighty-five human mandibular premolars were selected. Specimens were shaped to TruShape 25/.06 and divided into 5 groups (1 control and 4 test groups) according to the final activation technique (EndoActivator, EA), Ultrasonic (EndoUltra, PUI) and Laser (PIPS and SWEEPS). EDTA (Ethylenediaminetetraacetic acid) followed by NaOCl (Sodium Hypochlorite) and again EDTA were activated for each test group. Specimens were then split longitudinally and observed by Field Emission Scanning Electron Microscopy (FESEM). Blinded evaluation of the presence of smear layer was performed at 1000X magnification, according to a 5-score index system. Comparison between groups were analysed statistically using the Kruskal–Wallis non-parametric analysis of variance. Bonferroni multiple comparison tests were used.

**Results:**

At 1 mm only PIPS and SWEEPS performed better than the control group. At 3, 5 and 8 mm from the apex, every activation technique showed statistically significant reduction of smear layer when compared to the control group. PIPS and SWEEPS obtained better cleanliness result compared to EA, while only PIPS was superior to PUI in terms of cleanliness.

**Conclusions:**

PIPS and SWEEPS showed the best results in conservative canal preparations. Nowadays, contemporary rotary instruments allow fast and minimally invasive shaping of the endodontic space. In this scenario irrigants’ activation may be regarded as a mandatory step to a favourable clinical outcome.

## Background

One of the most important steps to endodontic success is the thorough instrumentation of the root canal space [1]. Root canal shaping of the endodontic system produces a smear layer, which consists of organic and inorganic substances [2]. The persistence of smear layer after root canal instrumentation may lead to several biological and physical shortcomings [3, 4]. Therefore, to dissolve both its organic and inorganic component, a post-shaping alternating irrigation with a calcium-chelating agent, such as EDTA and a deproteinizing agent, as NaOCl, is recommended [3–6]. To enhance the distribution and effectiveness of such irrigants, several activation techniques are currently being used. EndoActivator (EA) (Dentsply Tulsa Dental Specialties, Tulsa, OK) is a sonic handpiece that uses flexible non-cutting polymer tips to vigorously agitate intracanal irrigants [7]. Passive ultrasonic irrigation (PUI) relies on the acoustic streaming and cavitation effects, produced by an oscillating ultrasonically driven non-active file, to transport irrigants into the root canal system [8, 9]. EndoUltra (Vista, Racine, Wisconsin, USA) is a PUI cordless ultrasonic handpiece that features a smooth NiTi activator tip (15/0.02). Laser activation of irrigants has been introduced in addition to the traditional procedures [10, 11]. Photon-induced photo acoustic streaming (PIPS) is a laser agitation technique, which uses an erbium:yttrium–aluminum-garnet (Er:YAG) laser at 2940 nm (LightWalker AT; Fotona, Ljubljana, Slovenia). This technique is based on the high absorption of Er:YAG laser wavelength into water-based irrigants that fill the pulp chamber. When an Er:YAG laser is shot in aqueous medium, the irrigants are locally and instantly heated beyond their boiling point and a vapor bubble starts to form at the fiber tip’s end after each pulse [11]. This vapor bubble collapses after reaching its maximum volume with a subsequent cavitation effect. This phenomenon produces turbulent photoacoustic agitation of irrigants streaming the fluids three-dimensionally throughout the root canal system and leads to effective removal of smear layer [11]. SWEEPS (Shock Wave Enhanced Emission Photoacoustic Streaming) is a more recent Er:YAG laser modality launched to improve the cleaning and disinfecting efficacy of PIPS technique. It is based on the emission of a couple of consecutive laser pulses, with the second subsequent laser pulse that shoots into the liquid at an optimal delay time from the first pulse, when the initial bubble is in the final phase of its collapse. This phenomenon produces an acceleration of the laser-induced bubbles’ collapse, leading to the emission of shock waves also in narrow root canals [12]. As opposed to the conventional laser activated irrigation (LAI) procedure, which needs a certain canal enlargement to allow the laser tip to reach the apical portion of the root, PIPS and SWEEPS techniques only require the tip to be placed into the coronal reservoir of the pulp chamber. This allows for minimally invasive endodontic preparation [13]. However, to date, very little data is available regarding smear layer removal in canals that were shaped keeping narrow apical diameters [11, 14, 15], and no study has yet tested SWEEPS in relation to the smear layer removal. Therefore, the aim of this study was, to compare the smear layer removal, at 1, 3, 5 and 8 mm from the apex, after final irrigant activation with EA, PUI, PIPS and SWEEPS in conservatively shaped canals. The null hypothesis tested was that there is no difference in smear layer removal by using different final irrigant activation protocols.

## Methods

### Root canal preparation and irrigants’ activation procedures

Eighty-five single-rooted (mandibular premolars) teeth extracted for orthodontic reasons from young adult patients between 15 and 25 years old, were selected with the approval of the Ethics in Research Committee of the Centre of Health Sciences of the University of Rome “Tor Vergata”, Italy. Teeth were devoid of caries, cracks, endodontic treatments and restorations. Only teeth with intact and mature root apices and similar length (20–22 mm) were selected. Teeth were then radiographed bucco-lingually and mesio-distally. Teeth with root canal curvatures greater than 5° or calcified root canals were excluded. The degree of root canal curvature was calculated according to the Schneider’s method [16]. After extraction, teeth were stored in a solution of 2% thymol in distilled water at room temperature and used within one week. Inclusion and exclusion criteria were verified under a 20 × magnification laboratory microscope (Stemi DV4 Spot; Carl Zeiss, Oberkochen, Germany). After the access cavity was created, a #10 K-file (Dentsply Maillefer, Ballaigues, Switzerland) was inserted into the canal until the instrument tip was barely visible at the apical foramen. Specimens that allowed the introduction of an instrument exceeding ISO size 20 to the apical foramen were not included. The root lengths were standardized to 20 mm by grinding of the crowns perpendicular to the long axis by means of a high-speed, water-cooled diamond disc. To simulate clinical conditions, thus creating a closed-end environment, apices were sealed with composite after etching and bonding of the root surfaces. To prevent bonding and composite from entering the canal, a #10 K-file was inserted before the apex was sealed. The Pro-Train (Simit Dental, Mantova, Italy) was used to standardize the procedures for tooth preparation. Specimens were randomly divided in a control group (n = 5, no post-shaping irrigants activation procedures) and 4 experimental groups (n = 20). Groups were all shaped with TRUShape following the manufacturer’s recommendation (300 rpm, 3 N-cm). A gentle in-and-out pecking motion of about 2–3 mm in amplitude with light apical pressure was applied to the instrument until it reached the working length (WL). #25/0.06 was chosen as final instrument. Each instrument was used to shape only 3 specimens. After each instrument, canals were rinsed with 3 mL 5.25% NaOCl (Chematek Spa, Rome, Italy) at 37 °C. Each group was then rinsed with 10 mL of distilled water and, subsequently, irrigated with 3 ml 17% EDTA (Chematek Spa) and left in the canal for 1 min before being rinsed again with 10 mL distilled water and, subsequently, with 3 mL 5.25% NaOCl at 37 °C. Specimens were again rinsed with 10 mL of distilled water. A 30-G syringe needle (NaviTip; Ultradent, South Jordan, UT), was used to deliver irrigants. Then, specimens of the 5 groups underwent the following post-shaping irrigants activation procedures (Tables 1, 2). Irrigants were continuously delivered with a 30-G syringe needle, kept in the pulp chamber, at a 10 ml/min rate during activation for every activation technique. This is mandatory for PIPS and SWEEPS to maintain hydration and was also done for EA and PUI for standardization purpose.

**Table 1:** Post-shaping irrigation procedures

Group name	n	Shaping	Irrigation protocol	Activation
Control group (CTR)	5	Yes	–	No
EA–PUI–PIPS–SWEEPS	20 per group	Yes	30 s. 17% EDTA activation 30 s. distilled water activation 30 s. 5.25% NaOCl activation + 30 s. resting time 30 s. 5.25% NaOCl activation + 30 s. resting time 30 s. distilled water activation 30 s. 17% EDTA activation 30 s. distilled water activation	Yes

**Table 2:** Irrigants activation protocols

Group name	n	Shaping	Activation	Activation protocol
Control group (CTR)	5	Yes	No	–
EA	20	Yes	Yes	A 15/.02 tip was used at 2 mm from the WL with 2–3 mm of vertical excursions. The power setting was 10,000 cpm. The EndoActivator handpiece (Dentsply Tulsa Dental Specialties, Tulsa, OK) was used
PUI	20	Yes	Yes	A 15/.02 tip was used at 2 mm from the WL with 2–3 mm of vertical excursions. The oscillation frequency was 40 kHz (40,000 cps). The Endo Ultra (Vista dental products) was used
PIPS	20	Yes	Yes	A 2940 nm wavelength Er:YAG laser (Fidelis; Fotona, Ljubljana, Slovenia) with a 12 mm long, 400 µm quartz tapered tip was used. The tip had 3 mm of the polyamide sheath stripped back from its end. The laser parameters were as follows: 20 mJ per pulse, 15 Hz, 0.30 W and 50 µs pulse duration. The coaxial water spray of the handpiece was set to “off”. The tip was placed into the coronal access opening of chamber only, kept stationary and not advanced into the orifice of the canal
SWEEPS	20	Yes	Yes	A 2940 nm wavelength Er:YAG laser (Fidelis; Fotona, Ljubljana, Slovenia) with a flat-end fiber tip (SWEEPS 600 by Fotona) was used. The laser parameters were as follows: 40 mJ per pulse, 15 Hz, 0.30 W and 50 µs pulse duration. The coaxial water spray of the handpiece was set to “off”. The Auto SWEEPS laser modality was used. The tip was placed into the coronal access opening of chamber only, kept stationary and not advanced into the orifice of the canal

### Specimen preparation

Field emission scanning electron microscopy (FESEM) was used to evaluate endodontic smear layer removal from the shaped root canals. To facilitate fracture into halves, all roots were grooved longitudinally on the external surface with a diamond disc without penetration into the root canals. Specimens were then split into halves with a chisel, using a properly fitting gutta-percha cone in the root canal to limit tooth fragments covering endodontic canal walls. For each root, the half containing the most visible part of the endodontic wall was conserved in distilled water and coded. The coded specimens were secured on metal stubs, desiccated and viewed with FESEM (SUPRA 35; Carl Zeiss SMT, Oberkochen, Germany). The main operating parameters were 5 kV as gun voltage and a working distance of about 11 mm. Each specimen was first examined at low magnification (25 ×) for a general view of the canal. The magnification was then adjusted to 1000 ×, and four secondary electron (SE) micrographs (one for each canal level objective of the study: 1, 3, 5 and 8 mm from the apex) were taken in the same position at the approximate centre of the main shaped lumen of the canal with no attempt to select images showing any particular feature, such as open tubules. All procedures were performed by the same operator for standardization purpose.

### FESEM evaluation

**Fig. 1:**
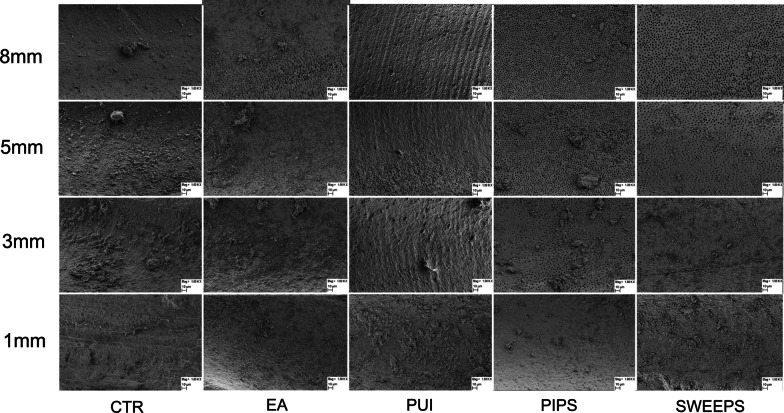
1000X micrographs of activation group specimens taken at 1,3,5 and 8mm from the apex

Cleanliness was evaluated by micrographs taken at 1, 3, 5 and 8 mm from the apex at 1000X magnification (Fig. 1). Two observers performed blind evaluation independently after examining 20 specimens for calibration purposes. Intra and inter examiner reliability for field emission scanning electron microscopic assessment was verified by the Kappa test. Cleanliness was evaluated according to a 5-score index system codified by Hulsmann [17], which measured the presence, quantity and distribution of the smear layer as follows: score 1 = no smear layer (dentinal tubules open), score 2 = small amount of smear layer (some dentinal tubules open), score 3 = homogeneous smear layer covering the root canal wall (only a few dentinal tubules open), score 4 = complete root canal wall covered by homogeneous smear layer (no open dentinal tubules), and score 5 = heavy non homogeneous smear layer covering the complete root canal wall.

### Statistical analysis

Comparison between groups were analysed statistically using the Kruskal–Wallis non-parametric analysis of variance. Bonferroni multiple comparison tests were used; P values were computed and compared with statistical significance at the P = 0.05 level. The data were analysed with the statistical software STATA (STATA Statistical Software Release 12.1; Stata Corp, College Station, TX).

## Results

**Fig. 2:**
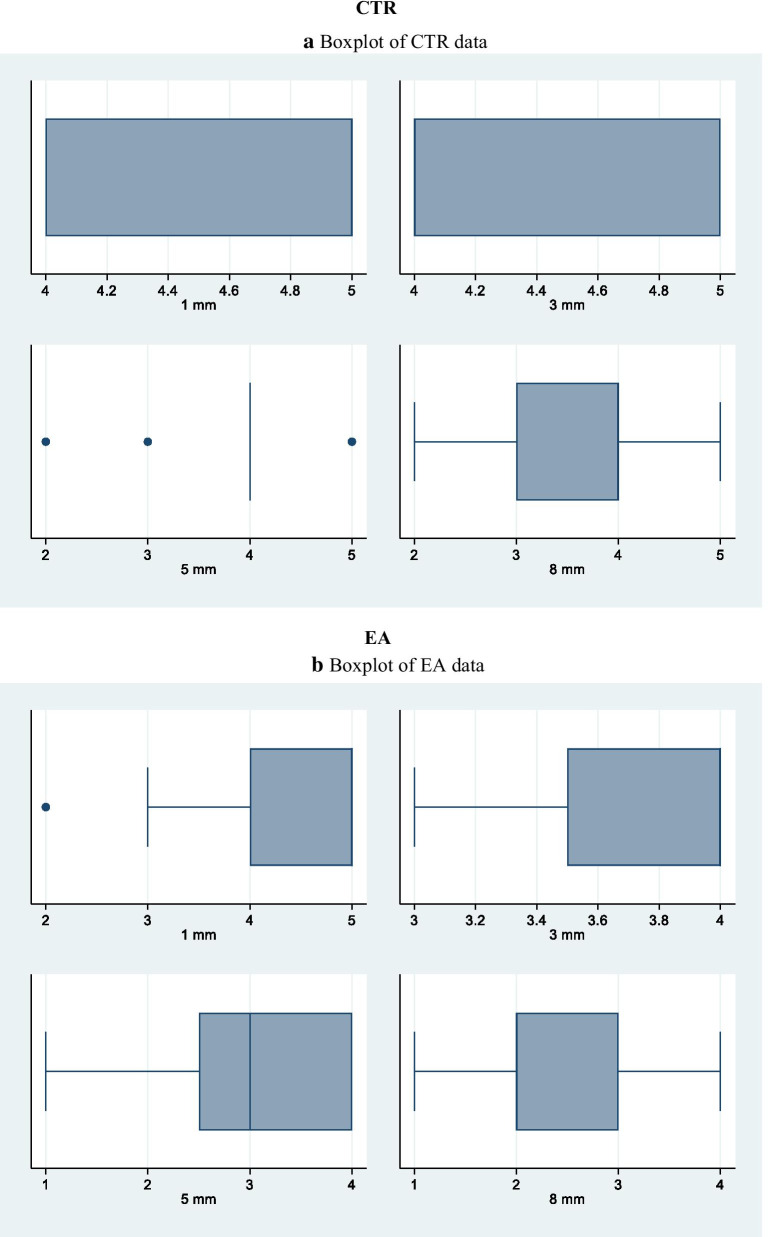

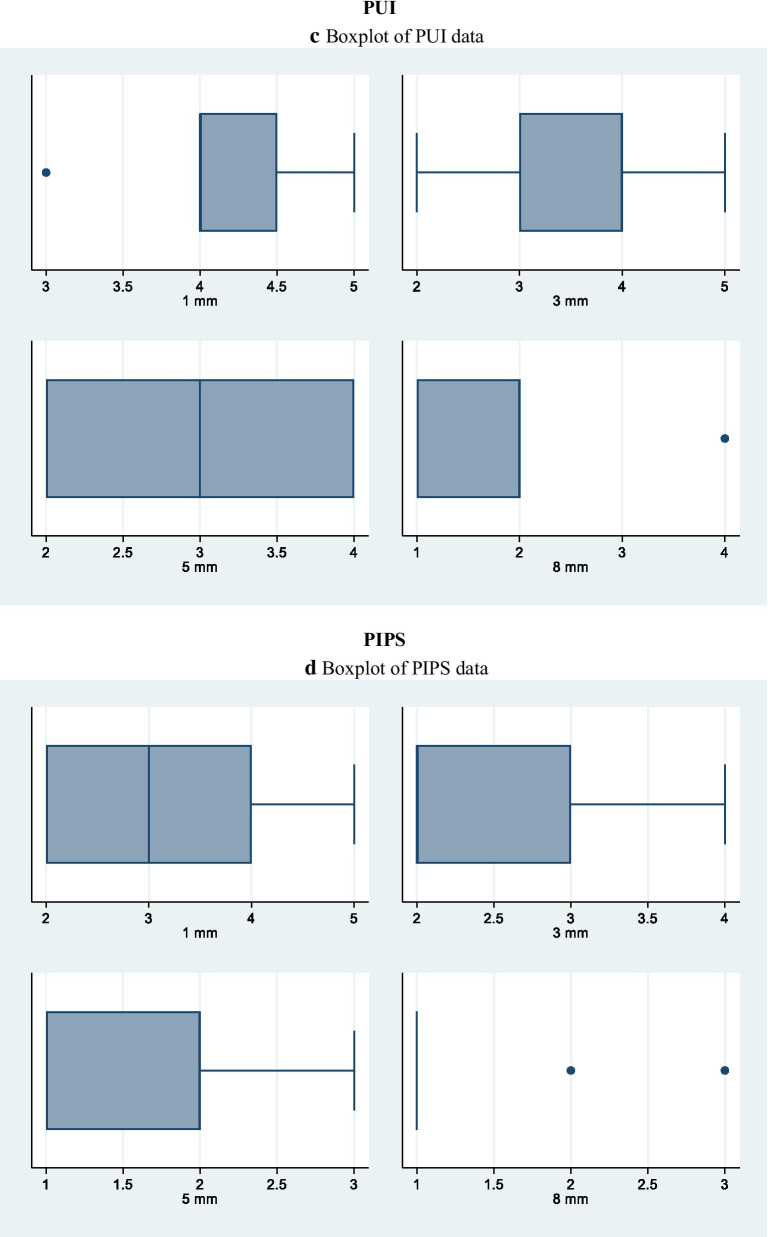

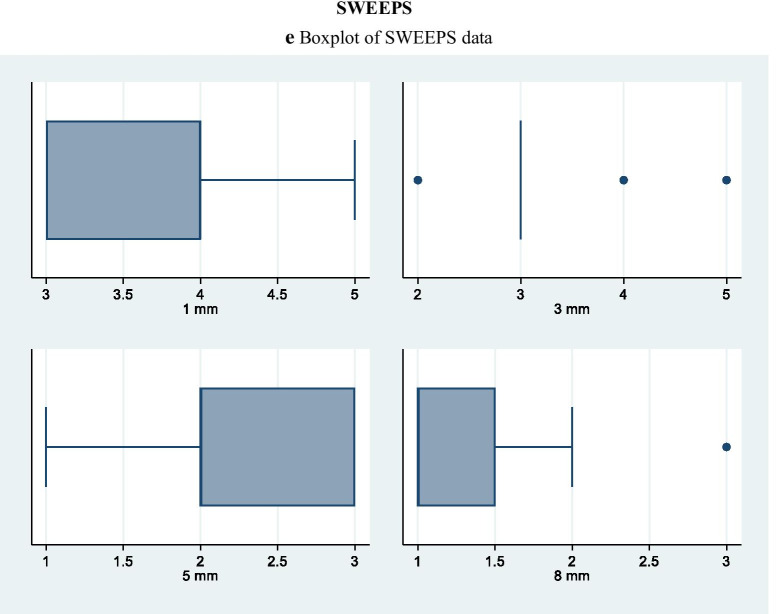
Boxplot of **a** CTR data, **b** EA data, **c** PUI data, **d** PIPS data, **e** SWEEPS data

Kappa test results, with a significance set at 0.5, showed good intra- and inter-examiner agreement, with values of 0.90 and above for the different groups. Cleanliness was evaluated analysing the field emission scanning electron microscopic photomicrographs. Table 3 shows comparison of the tested irrigation regimens on the cleaning efficacy at different distances from the apex. At 1 mm from the apex, the dentin surface was covered by heavy and irregular deposits of smear layer with dentinal tubules rarely visible with the exception of samples in which irrigants were activated with PIPS and SWEEPS. In fact, these techniques were the only ones that, at this level of the canal, performed statistically significantly better than the control group (35% of both specimens showed a score 3) and better than the control group and PUI group (30% of PIPS specimens showed a score 2). At 1 mm the smear layer removal ability of SWEEPS and EA were comparable. At 3, 5 and 8 mm from the apex all activation techniques showed statistically significant reduction of smear layer when compared to the control group. PIPS and SWEEPS reached better cleanliness results compared to EA, while only PIPS was superior to PUI in terms of cleanliness. All groups showed increased smear layer removal, moving apically to coronally (Table 3). At any distance from the apex no significant differences were found between EA and PUI and between PIPS and SWEEPS. Overall, PIPS revealed to be more efficient than SWEEPS in terms of smear layer reduction, even though, in terms of statistics, their results were comparable (Table 3 and Fig. 2a–e).

**Table 3:** Cleanliness of root canal treated with different methods expressed as Score percentages

	SCORE	1 (%)	2 (%)	3 (%)	4 (%)	5 (%)	1 (%)	2 (%)	3 (%)	4 (%)	5 (%)
		1 mm					3 mm				
A	CTR	0	0	0	44	56	0	0	0	67	33
B	EA	0	5	15	25	55	0	0	25	75	0
C	PUI	0	0	5	70	25	0	5	35	55	5
D	PIPS	0	30	35	30	5	0	55	40	5	0
E	SWEEPS	0	0	35	55	10	0	15	70	10	5
	Significant result at: p < 0.01		A,C vs D	A,C vs D,E	C vs D	A vs D,E B vs D		A,B,C vs D	A vs B,D,E B,C vs E	A,B,C vs D,E	A vs B,D
		5 mm					8 mm				
A	CTR	0	11	11	56	22	0	11	22	56	11
B	EA	5	20	45	30	0	15	45	25	15	0
C	PUI	0	35	35	30	0	30	65	0	5	0
D	PIPS	40	50	10	0	0	80	15	5	0	0
E	SWEEPS	20	40	40	0	0	75	20	5	0	0
	Significant result at: p < 0.01	A,B,C vs D	A vs D	A vs E B vs D	A,B,C vs D,E		A vs B, D,E B,C vs D,E	A vs B,C C vs D,E B vs D		A vs B,C,D,E	
	Significant result at: p < 0.05			A vs B	A vs C

## Discussion

Effective chemo-mechanical preparation of the root canal space is of paramount importance for a successful root canal treatment [1]. Nonetheless, shaping procedures can leave residual smear layer on dentinal walls [2], which may have a role in endodontic failure [3, 4]. Another crucial aspect to consider for the long-term success is dentin preservation [18]. Therefore, a challenge for present endodontics is to provide enhanced cleanliness efficiency while saving as much restorable tooth as possible. This in vitro study aimed the evaluation of the effectiveness of sonic (EndoActivator), ultrasonic (EndoUltra) and two lasers irrigant activation techniques (PIPS and SWEEPS) on the smear layer removal from the apex to the coronal third in minimally instrumented canals. Smear layer is usually removed by irrigants able to dissolve both its organic and inorganic components [6]. In the present study EDTA followed by NaOCl was used as it is currently considered the most effective irrigating clinical protocol [4–6]. Attempts were made to limit variables as much as possible. In fact, it was possible to standardize volume, rate, time of irrigation and irrigants activation. During irrigant activation, root canals were continuously replenished to maintain constant irrigants level [13]. A continuous flow of irrigants provides the advantage of a constant replacement of irrigant fluid [10] and it is crucial for the success of LAI techniques [19]. In contrast with previously published studies [20–22], in our investigation, canal shaping was kept conservative (apical size #25, taper 0.06), in order to save as much dentinal tissue as possible. Considering present conditions, PUI may result ineffective. In fact, it was suggested that small canal shapes may hinder its efficacy, as this technique relies on the ability of the activated instrument to oscillate freely, while when this does not happen, the acoustic streaming can lose intensity [9]. Nonetheless, in the present investigation there were no significant differences between PUI and SWEEPS efficacy at 1, 3, 5 and 8 mm from the apex, even though SWEEPS obtained better scores. This may be explained by the small EndoUltra tip size (15/0.02) used. In fact, the thinner the vibrating instrument, the higher the frequency. This leads to a higher streaming speed and a more intense acoustic streaming [9]. For standardization purpose, in the present study, a 15/0.02 tip was also selected for EA activation, as the 15/0.02 EndoUltra tip is the only one currently available on the market. Sonic activation operates at a lower frequency than ultrasonic activation. The positive relationship between frequency and streaming velocity should justify a better efficiency of PUI versus sonic activation [9]. Conversely, in our study, the differences between PUI and EA scores were small, though favouring PUI activation. In fact, no statistically significant differences were noticed between EA and PUI at any distance, but both the techniques removed significantly more smear layer at 3, 5 and 8 mm from the apex compared to the control group. This was coherent with previous papers [6, 20]. Conversely, Uroz-Torres et al. reported EA to remove no significant more smear layer than conventional irrigation [23]. These results might be attributed to the lower volume of irrigant and the shorter time of irrigation used compared to the present study [24]. Schmidt et al. showed that PUI did not remove more smear layer than conventional syringe irrigation [24], and this may be also attributed to the lower NaOCl volume and concentration used. Rödig et al. showed that EA obtained even better cleanliness result than ultrasonic activation [5]. This may be justified by the choice to use a K-file, instead of a blunt tip, for ultrasonic activation, which could have itself produced new undesirable smear layer. Similar results were already described, in which EA removed significantly greater smear layer than PUI at 3 mm from the apex [20, 22]. This could be explained by the choice to increase the apical shaping to a ProTaper F4 (apical size 0.40, taper 6% in the last 3 mm) in order to improve the volume exchange of irrigants. Conversely, PIPS and SWEEPS techniques seem to require no particular canal enlargement [13, 25]. Cavitation bubbles reach the bottom of the root canals even when the laser tip is maintained without walls contact in the access cavity, allowing canals to be shaped to considerably smaller sizes [11, 25]. Nonetheless, the question of ideal canal size and taper has yet to be addressed [25]. Peeters et al. suggest that it is possible to minimize the invasiveness of the treatment when using LAI, as in their study promising results were obtained in the apical region with minimal canal enlargement [14]. Laser activated irrigation using PIPS has been shown to be effective in significantly better cleaning of the root canal walls in comparison with conventional irrigation procedures [11, 21, 26, 27]. To the best of our knowledge, only a few studies compared PIPS with sonic or ultrasonic activation in terms of smear layer removal [21, 27]. Akyuz et al. showed how PIPS and PUI obtained similar cleanliness results [27]. Similarly, Arslan et al. found no significant difference between PIPS and EA in terms of cleanliness in the middle third of the canal [21]. These findings may be mainly explained by the larger canal shaping (ProTaper F4 as final instrument) used that could have facilitated EA and PUI action. In the present study PIPS was significantly more efficient than control group, EA and PUI at 1, 3, 5 and 8 mm from the apex. As mentioned before, PIPS and PUI efficacy is based on acoustic streaming and cavitation effects [9, 11]. These phenomena have an important role in the smear layer removal. In fact, PIPS has been found to be effective even when the activated solution was saline, which alone does not affect the smear layer [28]. Accordingly, in the present study, the choice to leave irrigants for 30 s of resting time as part of the post-shaping activation protocol was justified by the intention to rely also on their chemical action. SWEEPS has been developed in order to improve PIPS efficacy even further, aiming at producing shock waves even in spatially confined reservoirs [12]. Despite that, in the present study, there were no statistically significant differences between PIPS and SWEEPS at any distance from the apex. Nonetheless SWEEPS never removed significantly more smear layer than PUI, while it was always statistically more effective than control group and EA. The present study is the first one investigating SWEEPS in terms of smear layer removal. Therefore, it was not possible to discuss our findings with other reports, and we can only speculate that SWEEPS performance has been influenced by the present experimental conditions that, somehow, have affected its efficacy. Indeed, SWEEPS should be further tested in the future. This study has several limitations. Traditional SEM investigations have been reported to be not trustworthy. In fact, longitudinal observations, in which a given dentin area can be observed at different times, are to be considered as a more reliable study model [29]. Despite that, SEM evaluations are still the most used in research [2, 6, 11, 14, 20–22, 30] and, an ideal experimental model to assess smear layer removal seems not to be available at the moment [29]. An ideal experimental setting should also take into consideration the amount of sclerotic dentin [29]. In this study samples were taken from young patients. This may limit the occurrence to mistake sclerotic dentin, which increases with aging, for smear-layer covered dentin. Another issue is the qualitative scoring method used, even though it is one of the most used in literature [2, 11, 14, 20–22]. Computational systems, able to automatically extract quantitative data, are likely to minimize human bias [29]. Nonetheless, in our investigation, the use of a direct qualitative scoring system, by multiple calibrated examiners with concordance between them (Kappa test), together with the large number of observations made, may considerably increase the reliability of the results [6, 8].

## Conclusions

Within the limitations of this study, it can be concluded that, in terms of smear layer removal, PIPS guarantees the best results in conservative root canal preparations. The differences with control, EA and PUI groups are significant especially in the apical third, that can be seen as a critical area for the outcome of endodontic therapies. SWEEPS also scored better than EA and PUI, but the differences with PUI were never significant and, compared to EA, significance was registered only at 3, 5 and 8 mm from the apex. Therefore, the null hypothesis has to be rejected. Furthermore, nowadays, more conservative NiTi rotary instruments are available. Thus, further studies in even narrower canals, will be needed in the future to integrate our findings.

## Data Availability

The datasets used and/or analysed during the present study are available from the corresponding author on reasonable request.

## Abbreviations

EA: EndoActivator
PUI: Passive ultrasonic irrigation
PIPS: Photon induced photoacoustic streaming
SWEEPS: Shock wave enhanced photoacoustic streaming
EDTA: Ethylenediaminetetraacetic acid
NaOCl: Sodium hypochlorite
FESEM: Field emission scanning electron microscope
ErYAG: Erbium:yttrium–aluminum-garnet
LAI: Laser activated irrigation
SE2: Second electron detector
